# Astrocyte/monocyte activation and inflammation are associated with asymptomatic neurocognitive impairment in people with HIV on suppressive antiretroviral therapy

**DOI:** 10.1097/QAD.0000000000004534

**Published:** 2026-05-08

**Authors:** Matteo Augello, Alessandra Mingione, Lidia Borghi, Andrea Corona, Valeria Bono, Roberta Rovito, Valentina Sala, Martina Giudice, Camilla Tincati, Elena Vegni, Filippo Martinelli Boneschi, Giulia Marchetti

**Affiliations:** a Clinic of Infectious Diseases and Tropical Medicine; b Clinic of Neurology; c Clinical Psychology Unit, San Paolo Hospital, ASST Santi Paolo e Carlo, Department of Health Sciences, University of Milan, Milan, Italy.

**Keywords:** astrocyte activation, gut barrier disruption, HIV-associated neurocognitive disorder, inflammation, microbial translocation, monocyte activation, neuroaxonal damage

## Abstract

**Objective:**

Despite suppressive antiretroviral therapy (ART), people with HIV (PWH) frequently experience neurocognitive impairment, yet underlying mechanisms remain elusive. We hereby evaluated biomarkers of brain injury, inflammation and gut barrier dysfunction to identify factors associated with cognitive performance in PWH.

**Design:**

PWH on suppressive ART and people without HIV (PWoH) were cross-sectionally enrolled.

**Methods:**

PWH underwent a comprehensive neurocognitive assessment and HIV-associated neurocognitive disorder (HAND) was diagnosed. We measured plasma markers of neuroaxonal damage and astrocyte activation (NF-L/GFAP by Simoa), inflammation/monocyte activation (IP-10/IL-6/sCD14 by ELISA), gut barrier disruption (I-FABP/E-cadherin/zonulin by ELISA) and microbial translocation (LBP/EndoCAb IgG by ELISA). Non-parametric tests and multivariable regression were used for analyses.

**Results:**

Forty PWH [17 HAND+ (asymptomatic neurocognitive impairment), 23 HAND–] and 10 PWoH were included. Compared to PWoH, PWH had higher levels of inflammation/monocyte activation (IP-10/sCD14), gut barrier disruption (I-FABP) and microbial translocation (LBP/EndoCAb-IgG). PWH HAND+ showed higher levels of GFAP/IL-6/sCD14 compared to PWH HAND–. At logistic regression adjusting for age, sex and CD4 T-cell *nadir*, HAND confirmed associated with higher GFAP/sCD14. More specifically, at linear regression adjusting for the same variables, higher GFAP levels were associated with lower global cognitive performance, worse attention and working memory, speed of information processing, motor skills, as well as learning and memory; higher IL-6 with worse abstraction and executive functions; higher IL-6 and sCD14 with worse verbal fluency.

**Conclusion:**

In our cohort of ART-suppressed PWH, asymptomatic neurocognitive impairment was associated with markers of astrocyte/monocyte activation and inflammation rather than with markers of neuroaxonal damage, gut barrier disruption, or microbial translocation.

## Introduction

Despite a substantial decline in the prevalence of severe HIV-associated neurocognitive impairment in the antiretroviral therapy (ART) era, people with HIV are still frequently affected by subtler forms [1,2]. Asymptomatic neurocognitive impairment, although not significantly interfering with daily activities by definition [3], is associated with worse quality of life [4] and a considerably higher risk of developing symptomatic cognitive decline [5], thus underlining the need to recognize and follow up these individuals.

As comprehensive neurocognitive testing is cumbersome, time-intensive, and demands highly specialized personnel, blood-based biomarkers represent a promising tool for the screening of PWH who may require further evaluation, as well as for the close monitoring of individuals ultimately diagnosed with asymptomatic neurocognitive impairment. Moreover, such biomarkers may provide valuable insights into the neuropathogenesis of HIV-associated neurocognitive impairment, potentially guiding the development of targeted therapeutic strategies based on the underlying mechanisms.

Several factors have been involved in the pathogenesis of the HIV-associated neurocognitive impairment, including the dysregulation of the gut–brain axis, that is the bidirectional communication network between the gastrointestinal tract and the central nervous system (CNS) [6]. Gut barrier disruption and microbial translocation have long been acknowledged as hallmarks of HIV infection associated with persistent inflammation and immune activation as well as non-AIDS comorbidities despite effective ART [7]. Nonetheless, previous research exploring the role of these phenomena in the pathogenesis of neurocognitive impairment is scarce and mainly focused on ART-naive people with HIV [8] or on specific high-risk subgroups such as those with hepatitis C virus (HCV) coinfection or heavy drinkers [9,10], thereby limiting the generalizability of the findings to the more typical clinical scenarios in the current ART era.

In this context, the aim of present study was to assess possible associations between plasma biomarkers of brain injury, inflammation, and gut barrier dysfunction with cognitive performance in a cohort of virologically suppressed people with HIV.

## Methods

### Study design and population

In this cross-sectional study, people with HIV on suppressive ART and age/sex-matched healthy people without HIV were consecutively enrolled at the Clinic of Infectious Diseases and Tropical Medicine, San Paolo Hospital, ASST Santi Paolo e Carlo, Department of Health Sciences, University of Milan, Milan, Italy, between 2015 and 2019.

Inclusion criteria for people with HIV were: age at least 18  years, being virologically suppressed (plasma HIV-RNA <50  copies/ml) on ART, comprehension and speaking of Italian language. Exclusion criteria were: neurological/psychiatric diseases, previous CNS opportunistic infections, alcohol/substances abuse, cirrhosis.

Peripheral blood was collected in EDTA tubes; plasma was separated by centrifugation and stored at –80 °C. People with HIV underwent a comprehensive neurocognitive assessment by a trained neuropsychologist, and the Instrumental Activities of Daily Living (IADL) scale was used to assess daily living autonomy. Demographic and HIV-related data were also collected.

The study was approved by the Ethics Committee Milano Area 1 (no. 476, 06/04/2018), and written informed consent was obtained from each participant.

### Neurocognitive assessment

The neurocognitive evaluation included several tests exploring six cognitive domains: attention and working memory, learning and memory, abstraction and executive functions, speed of information processing, motor skills, verbal fluency (Supplementary material).

Based on demographically adjusted neurocognitive tests scores and IADL score, participants were diagnosed with HIV-associated neurocognitive disorder (HAND) as defined by the Frascati criteria [3].

Additionally, raw scores from each test were converted into standardized *T*-scores (Supplementary material). Domain-specific composite *T*-scores were then calculated by averaging the *T*-scores of the individual tests defining the specific cognitive domain. The global cognitive composite *T*-score was then obtained by averaging the domain-specific composite *T*-scores. Higher *T*-scores indicate better cognitive performance.

### T-cell phenotypes

CD4^+^ T cells (CD4^+^), CD8^+^ T cells (CD8^+^), functionally-competent CD4^+^ T cells (CD4^+^CD127^+^), activated CD8^+^ T cells (CD8^+^CD38^+^), naive CD4^+^ T cells (CD4^+^CD45RA^+^), and naive CD8^+^ T cells (CD8^+^CD45RA^+^) were quantified in people with HIV on fresh whole blood by flow cytometry (Supplementary material, Figure S1).

### Plasma biomarkers

Biomarkers of neuroaxonal damage (NF-L) and astrocyte activation (GFAP) were measured by Quanterix Simoa Neurology 2-Plex B Kit on the Quanterix SR-X Biomarker Detection System. Biomarkers of inflammation (IP-10, IL-6), monocyte activation (sCD14), gut barrier disruption (I-FABP, E-cadherin, zonulin) and microbial translocation (LBP, EndoCAb IgG) were measured by ELISA using commercial kits (Supplementary material).

### Statistical analysis

Categorical variables were presented as numbers (percentages), while continuous variables as medians (interquartile ranges). Given the non-normal distribution of the data, nonparametric tests were used for the statistical analysis. In detail, demographic and HIV-related characteristics as well as T-cell phenotypes were compared between people with HIV with HAND (HAND+) and without HAND (HAND–) by Mann–Whitney test (continuous variables) and Fisher exact test (categorical variables). Comparisons of plasma biomarkers between people with HIV HAND+, people with HIV HAND–, and people without HIV as well as comparisons of biomarkers according to the ART regimen were performed by Kruskall–Wallis test; Dunn's multiple comparisons test was used to account for multiple comparisons. Correlations between biomarkers were evaluated by Spearman's correlation test in the whole study population including both people with and without HIV. The association of each biomarker (independent variable) with the HAND status (dependent variable) in people with HIV was analyzed by logistic regression, both unadjusted and adjusted for age, sex, and CD4^+^ T-cell *nadir*. The association between each biomarker (independent variable) and global or domain-specific composite *T*-scores (dependent variables) was analyzed by both Spearman's correlation and linear regression analysis, also unadjusted and adjusted for age, sex, and CD4^+^ T-cell *nadir*. Analyses were performed by GraphPad Prism 10.4.2, with significance set at *P* < 0.05.

## Results

Forty people with HIV and 10 people without HIV were recruited. Seventeen people with HIV were diagnosed with HAND (asymptomatic neurocognitive impairment, ANI), while 23 were not. Demographic and HIV-related characteristics of people with HIV overall and according to HAND status are detailed in Table 1. Briefly, median age was 47 [interquartile range (IQR) 39–58] years, 30 (75%) were males, and 38 (95%) were Caucasian. Median CD4^+^ T-cell count was 608 (IQR 458–810) cells/μl, with a median CD4^+^/CD8^+^ ratio of 0.80 (IQR 0.56–1.09). They were on ART for a median of 38 (IQR 10–72) months, and all of them were virologically suppressed. The most common ART regimens were NNRTI-based (47.5%), followed by PI-based (27.5%) and INSTI-based (20%) regimens. No differences were recorded in demographic/HIV-related characteristics or T-cell phenotypes according to HAND status, except for a lower CD4^+^ T-cell *nadir* in people with HIV HAND+. Consistent with the definition, people with HIV HAND+ had worse performances in all explored cognitive domains as compared to those HAND– (Figure S2a–f), with all domains-specific composite *T*-scores positively correlating to each other (Figure S2g).

**Table 1:** Demographic and HIV-related characteristics of people with HIV overall and according to HIV-associated neurocognitive disorder status.

Characteristics	All PWH (*n* = 40)	PWH HAND+ (*n* = 17)	PWH HAND– (*n* = 23)	*P* value^a^
Age, years [median (IQR)]	47 (39–58)	46 (38–59)	47 (40–55)	0.9622
Male sex [*n* (%)]	30 (75)	11 (64.7)	19 (82.6)	0.2743
Ethnicity [*n* (%)] Caucasian Latin Asian	38 (95) 1 (2.5) 1 (2.5)	15 (88.2) 1 (5.9) 1 (5.9)	23 (100) 0 (0) 0 (0)	0.1744
Epidemiology MSM MSW/WSM IDU MTCT	23 (57.5) 11 (27.5) 4 (10) 2 (5)	8 (47.1) 4 (23.5) 3 (17.6) 2 (11.8)	15 (65.2) 7 (30.4) 1 (4.4) 0 (0)	0.1786
Comorbidities [*n* (%)] None Hypertension Cardiovascular disease Cerebrovascular disease Chronic kidney disease Liver disease COPD/asthma Diabetes Previous lymphoma Previous solid tumur	20 (50) 8 (20) 5 (12.5) 1 (2.5) 2 (5) 1 (2.5) 4 (10) 3 (7.5) 1 (2.5) 2 (5)	7 (41.2) 3 (17.6) 3 (17.6) 1 (5.9) 0 (0) 0 (0) 2 (11.8) 3 (17.6) 1 (5.9) 1 (5.9)	13 (56.5) 5 (21.7) 2 (8.7) 0 (0) 2 (8.7) 1 (4.4) 2 (8.7) 0 (0) 0 (0) 1 (4.4)	0.5231 0.9999 0.6340 0.4250 0.4987 0.4250 0.9999 0.0688 0.4250 0.9999
Hepatitis virus coinfection [*n* (%)] HCV-Ab+/HCV-RNA– HBcAb+/HBsAg+ HBcAb+/HBsAg–	4 (10) 1 (2.5) 14 (35)	3 (17.6) 1 (5.9) 8 (47.1)	1 (4.3) 0 (0) 6 (26.1)	0.2941 0.4250 0.1978
Viro-immunologic parameters [median (IQR)] CD4^+^ T-cell *nadir* (cells/μl) CD4^+^ T cells (cells/μl) CD8^+^ T cells (cells/μl) CD4^+^CD127^+^ T cells (cells/μl) CD8^+^CD38^+^ T cells, cells/μl CD4^+^CD45RA^+^ T cells, cells/μl CD8^+^CD45RA^+^ T cells, cells/μl CD4^+^/CD8^+^ ratio HIV-RNA (copies/ml)	281 (158–353) 608 (458–810) 802 (653–1164) 386 (308–487) 50 (29–89) 192 (93–311) 431 (268–532) 0.80 (0.56–1.09) <50	182 (98–296) 521 (376–696) 896 (532–1170) 385 (272–438) 41 (22–96) 169 (84–236) 365 (244–508) 0.68 (0.47–1.04) <50	322 (217–379) 633 (485–883) 800 (667–1093) 390 (308–693) 55 (35–89) 228 (114–366) 431 (290–577) 0.85 (0.62–1.09) <50	0.0170 0.0996 0.9018 0.5393 0.4343 0.1469 0.3743 0.3118 0.9999
Previous AIDS diagnosis [*n* (%)]	4 (10)	1 (5.9)	3 (13)	0.6235
Time from HIV diagnosis, months [median (IQR)]	102 (69–218)	150 (84–279)	97 (52–126)	0.0539
Current ART regimen [*n* (%)] INSTI-based PI-based INSTI+PI-based NNRTI-based	8 (20) 11 (27.5) 2 (5) 19 (47.5)	3 (17.6) 3 (17.6) 2 (11.8) 9 (53)	5 (21.7) 8 (34.8) 0 (0) 10 (43.5)	0.3140
Duration of ART, months [median (IQR)]	38 (10–72)	43 (8–100)	26 (10–64)	0.8662

MSM, men who have sex with men; MSW, men who have sex with women; WSM, women who have sex with men; IDU, injective drug use; MTCT, mother-to-child transmission; COPD, chronic obstructive pulmonary disease; INSTI, integrase strand-transfer inhibitor; PI, protease inhibitor; NNRTI, non-nucleoside reverse transcriptase inhibitor; CD4^+^CD127^+^ T cells, functionally-competent CD4^+^ T cells; CD8^+^CD38^+^ T cells, activated CD8^+^ T cells; CD4^+^CD45RA^+^ T cells, naive CD4^+^ T cells; CD8^+^CD45RA^+^ T cells, naive CD8^+^ T cells.

^a^ Statistical analysis: Mann–Whitney test and Fisher exact test, as appropriate (PWH HAND+ *versus* PWH HAND–).

**Fig. 1:**
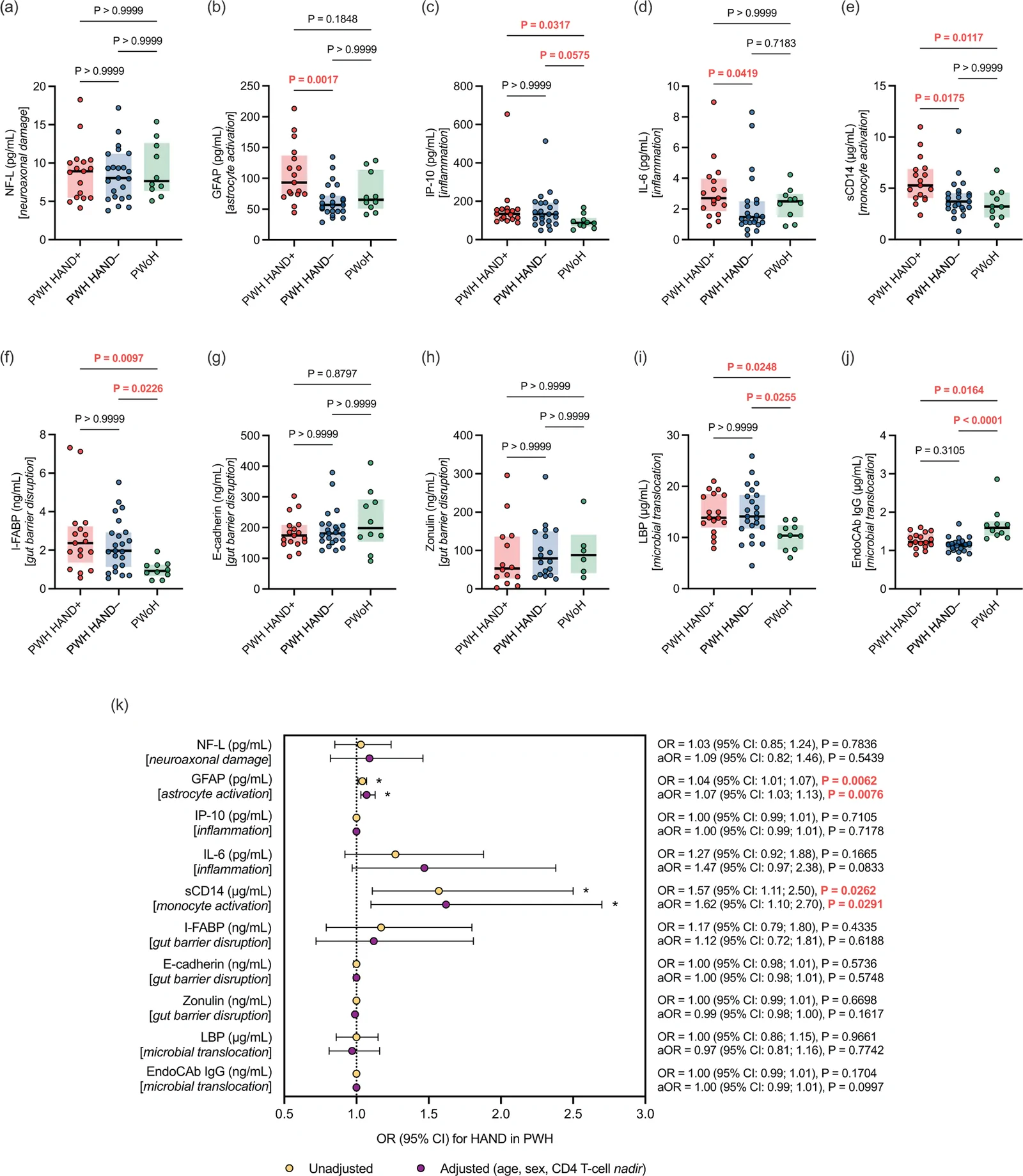
(a–j) Plasma biomarkers of neuroaxonal damage (NF-L), astrocyte activation (GFAP), inflammation (IP-10, IL-6), monocyte activation (sCD14), gut barrier disruption (I-FABP, E-cadherin, zonulin) and microbial translocation (LBP, EndoCAb IgG) in people with HIV (PWH) with HAND (*n* = 17), PWH without HAND (*n* = 23), and people without HIV (PWoH) (*n* = 10). Circles: individual values; black bars: median values; boxes: interquartile ranges; statistical analysis: Kruskall–Wallis test with Dunn's multiple comparisons test (*P* values <0.1 shown in red). (k) Association between plasma biomarkers and HAND in PWH (*n* = 40). Circles: odds ratio (OR) or adjusted odds ratio (aOR); error bars: 95% confidence interval (CI); statistical analysis: univariable and multivariable logistic regression (*P* values <0.05 shown in red). HAND, HIV-associated neurocognitive disorder.

As expected, people with HIV had higher levels of IP-10, sCD14, I-FABP and LBP, as well as lower levels of EndoCAb IgG, compared to people without HIV (Fig. 1c,e,f,i,j). The assessment of plasma biomarkers in people with HIV according to HAND status revealed elevated concentrations of GFAP, IL-6, and sCD14 in people with HIV HAND+ relative to those HAND– (Fig. 1b,d,e). By contrast, no differences in markers of neuroaxonal damage, gut barrier disruption and microbial translocation were recorded between people with HIV HAND+ and HAND– (Fig. 1a,f–j). Of note, no significant differences in the measured biomarkers were found according to the ART regimen in PWH (Figure S3).

When analyzing correlations between biomarkers across the whole study population to explore overall biological relationships among them (Figure S4), NF-L and GFAP appeared to be positively correlated to each other (ρ= 0.3373, *P* = 0.0166), as were I-FABP and LBP with IP-10 (ρ= 0.3452, *P* = 0.0141; ρ = 0.3315, *P* = 0.0187, respectively), and LBP with IL-6 (ρ= 0.3780, *P* = 0.0068). On the contrary, LBP and EndoCAb IgG were negatively correlated (ρ= –0.4100, *P* = 0.0031).

Then, a logistic regression analysis was performed to determine the association between biomarkers and HAND in people with HIV. Higher GFAP and sCD14 confirmed associated with HAND at both univariable and multivariable analysis adjusting for age, sex, and CD4^+^ T-cell *nadir* (Fig. 1k).

**Fig. 2:**
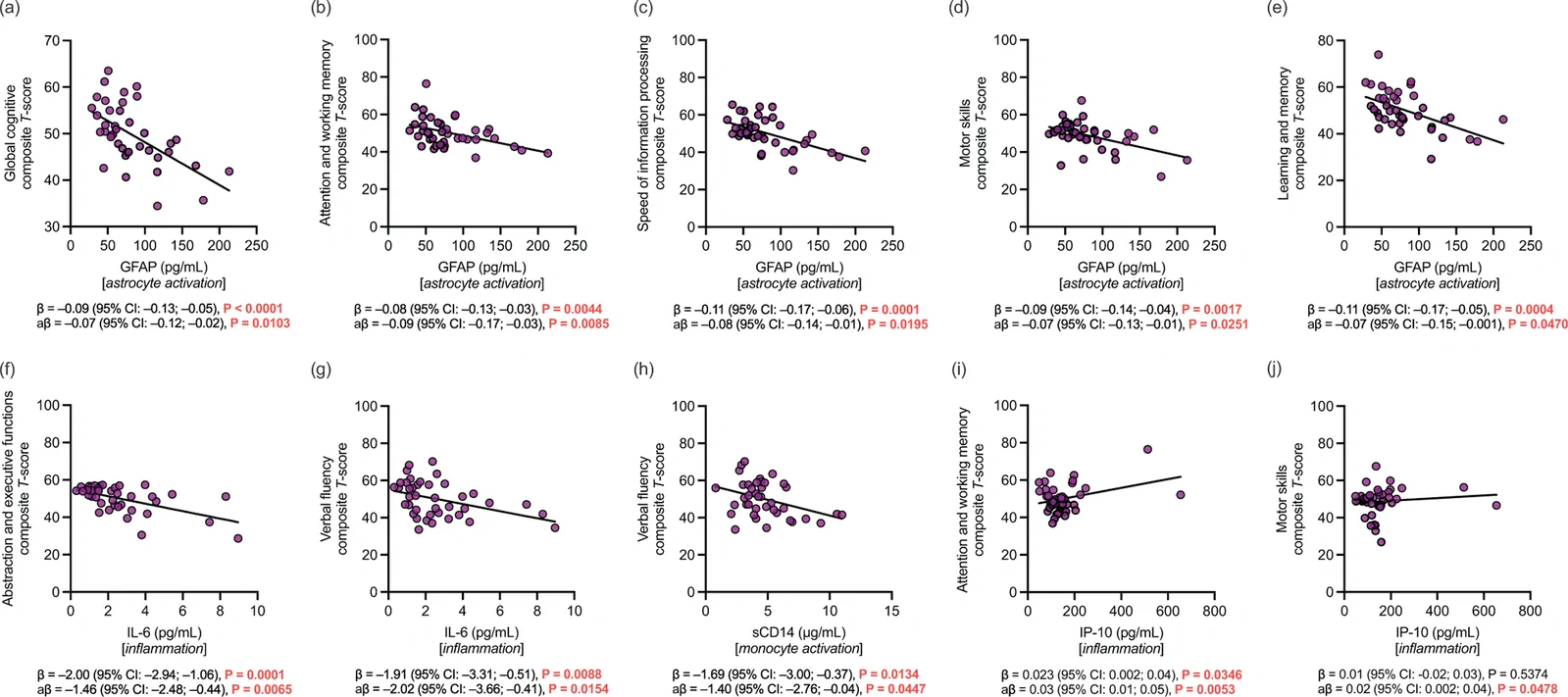
Association between plasma biomarkers and cognitive performances as measured by global cognitive composite *T*-score and domain-specific composite *T*-scores in people with HIV (*n* = 40). Circles: individual values; black line: fitted line. aβ, adjusted β coefficient; β, β coefficient; 95% CI, 95% confidence interval. Statistical analysis: univariable and multivariable (adjusted for age, sex, and CD4^+^ T-cell *nadir*) linear regression (only statistically significant associations are shown in the figure; see Table S2 for complete numerical data).

Lastly, several biomarkers were negatively correlated with worse performances across different cognitive domains as measured by domain-specific composite *T*-scores (Table S1). To further investigate these relationships, linear regression models adjusting for age, sex, and CD4^+^ T-cell *nadir* in the entire population of people with HIV were also fitted (Table S2): higher GFAP levels were associated with lower global cognitive composite *T* scores (Fig. 2a) and with worse performance in attention and working memory, speed of information processing, motor skills, as well as learning and memory (Fig. 2b–e); higher IL-6 with worse abstraction and executive functions (Fig. 2f); higher IL-6 and sCD14 with worse verbal fluency (Fig. 2g–h). Conversely, higher IP-10 was associated with better performance in attention and working memory as well as motor skills (Fig. 2i–j).

## Discussion

In this cross-sectional study, we assessed plasma biomarkers of brain injury, inflammation, and gut barrier dysfunction in a cohort of virologically suppressed people with HIV either with or without HAND (specifically, ANI) to identify biological factors associated with poor cognitive performance.

In keeping with previous literature [7], we confirmed that inflammation (IP-10, IL-6), monocyte activation (sCD14), gut barrier disruption (I-FABP), and microbial translocation (LBP, EndoCAb IgG) are hallmarks of HIV infection. Likewise, gut barrier impairment with ensuing microbial translocation (in turn associated with anti-endotoxin antibodies consumption) correlated with inflammatory biomarkers, supporting their role as important contributors to the HIV-associated systemic inflammation [7].

Conversely, markers of neuroaxonal damage (NF-L) and astrocyte activation (GFAP) did not differ between people with and without HIV. This is in accordance with prior studies showing similar levels of NF-L and GFAP in ART-treated people with HIV and people without HIV [11–14], yet in conflict with others [15].

Our finding of a significantly lower CD4^+^ T-cell *nadir* in people with HIV with HAND is consistent with previous literature showing that it is associated with an increased risk of neurocognitive impairment, even in individuals on virologically effective ART [1,16]. This observation supports the concept of a ‘legacy effect’, whereby severe immunosuppression occurring before treatment initiation may lead to long-lasting or irreversible CNS injury [17] and therefore neurocognitive impairment [18].

When assessing the measured biomarkers according to HAND status in people with HIV, we found that astrocyte/monocyte activation (GFAP, sCD14) and inflammation (IL-6) were associated with neurocognitive impairment. In keeping with these findings, higher GFAP levels were also associated with poorer global cognitive performance as measured by the global cognitive composite *T*-score. Inconsistent findings have been reported in prior research concerning the relationship between GFAP and cognition: while some studies found an association of this biomarker with poor cognitive function in people with HIV [19], others failed to show such a link [14,15]. Conversely, higher plasma levels of sCD14 have been invariably associated with HAND across several studies [20,21]; this finding support the hypothesis that monocyte activation contributes to neurocognitive impairment in people with HIV through the transmigration of activated monocytes across the blood–brain barrier, followed by infection and activation of resident brain cells and the production of neurotoxic factors that ultimately lead to neuronal injury [22]. Likewise, heightened levels of pro-inflammatory cytokines, including IL-6, have been described to feature HAND in several studies [8,20].

Notably, biomarkers of neuroaxonal damage (NF-L), gut barrier disruption (I-FABP, E-cadherin, zonulin), and microbial translocation (LBP, EndoCAb IgG) were not associated with HAND in our cohort. Previous studies exploring the association of plasma NF-L with cognitive performance in people with HIV yielded contradictory findings, with some of them reporting an association of higher levels of NF-L with worse cognition in ART-treated people with HIV [13,15,19], whilst others describing a similar association in ART-naive people with HIV but not in those on effective ART [12]. However, it must be noted that all people with HIV in our cohort were diagnosed with ANI, suggesting that substantial neuroaxonal injury sufficient to underlie more advanced neurocognitive impairment may not yet have occurred or otherwise may have reversed following ART initiation; this interpretation is further supported by the similar NF-L levels observed in people with and without HIV. Likewise, circulating LPS levels have been previously associated with severe HAND but not with ANI in people with advanced HIV disease [8], whilst in ART-treated people with HIV, neither I-FABP [23,24] nor LPS [23] were associated with objectively measured cognitive performances. Furthermore, LPS was reported as associated with HAND in people with HIV and HCV coinfection, but not in the HCV-negative ones [9], and with slower processing speed in heavy-drinking people with HIV [10], pointing to a potential involvement of the gut–brain axis in specific subgroups of people with HIV with additional risk factors. Interestingly, a more detailed analysis at the level of individual cognitive domains revealed that distinct biomarkers were selectively associated with impairment in certain domains. Specifically, GFAP was associated with worse attention, working memory, speed of information processing, motor skills, as well as learning and memory, sCD14 with reduced verbal fluency, whereas IL-6 with impaired abstraction, executive functions, and verbal fluency. Notably, these associations remained consistent after adjusting for age, sex, and CD4 T-cell *nadir* to account for demographic influences and potential legacy effects, suggesting that the links between biomarkers and cognitive performance are robust and largely independent of these factors. Overall, these findings suggest that distinct pathogenetic mechanisms – such as astrocyte/monocyte activation and inflammation – may differentially affect particular cerebral areas or certain inter-areal neuronal networks and thus contribute to the alteration of specific neurocognitive functions in people with HI

HIV.

Some limitations should be acknowledged in this study, including the small sample size that may hinder the generalizability of the findings, and the absence of mild/severe forms of HAND, that would have broadened the characterization of the HIV-associated neurocognitive impairment spectrum. Despite these limitations, our results provide exploratory insights that may inform future research in larger and more diverse cohorts.

In conclusion, these data suggest that markers of astrocyte/monocyte activation and inflammation are associated with asymptomatic neurocognitive impairment in ART-suppressed people with HIV, whereas no clear evidence emerged for neuroaxonal damage, gut barrier disruption, or microbial translocation in this cohort. Stemming from these findings are the potential application of such circulating proteins as early biomarkers of neurocognitive decline in people with HIV. However, further studies are needed to determine whether these associations reflect underlying pathogenic mechanisms, which would be necessary to support the potential exploitation of these pathways as therapeutic targets.

## Acknowledgements

We are grateful to all the individuals enrolled in this study who agreed to participate to this research. Our special thanks also go to all the physicians and nurses at the Clinic of Infectious Diseases and Tropical Medicine at San Paolo Hospital in Milan who helped in patients’ care and enrolment.

Authors contributions: M.A. conceived and designed the study, performed experiments, collected clinical data, analyzed and interpreted the data, designed the figures, and wrote the manuscript. A.M. performed part of the experiments. L.B. administered neurocognitive tests to people with HIV. A.C. contributed to data analysis. V.B., R.R., and V.S. contributed to data collection. C.T., E.V., and F.M.B. contributed to the critical revision of the manuscript. G.M. conceived and supervised the study, interpreted the data, and wrote the manuscript.

Funding: this study was supported by funding from the Italian Ministry of Health (‘Ricerca Finalizzata 2019’, RF-2019-12371089, CUP C44G19000060001) and by the Department of Health Sciences of the University of Milan (PSR2021_DIP_013).

### Conflicts of interest

There are no conflicts of interest.

## Supplementary Material

Supplemental Digital Content
