# Supplementary material for: Astrocyte/monocyte activation and inflammation are associated with asymptomatic neurocognitive impairment in people with HIV on suppressive antiretroviral therapy
Source: AIDS. 2026 May 8;40(9):1297–304. doi: 10.1097/QAD.0000000000004534 (PMC13566472; doi:10.1097/QAD.0000000000004534)
Supplement: Supplementary file 1 [file aids-40-1297-s001.pdf]

Supplementary material

Supplementary methods

Neurocognitive assessment

The neurocognitive evaluation administered to people with HIV included several tests exploring six main cognitive domains that are commonly affected in HIV-associated neurocognitive disorders (HAND)[1-5]: *attention and working memory* [Digit Span Test – Forward (DST-F), Digit Span Test – Backward (DST-B), Corsi Block Tapping Task (CBTT), Trail Making Test – part BA (TMT-BA)]; *learning and memory* [Rey Auditory Verbal Learning Test – Immediate Recall (RAVLT-IR), Rey Auditory Verbal Learning Test – Delayed Recall (RAVLT-DR), Rey-Osterrieth Complex Figure test – Delayed Recall (ROCF-DR)]; *abstraction and executive functions* [Rey-Osterrieth Complex Figure test – Copy (ROCF-C), Trail Making Test – part B (TMT-B), Stroop Colour and Word Test – Errors (SCWT-E)]; *speed of information processing* [Trail Making Test – part A (TMT-A), Stroop Colour and Word Test – Time (SCWT-T), Symbol Digit Modalities Test (SDMT)]; *motor skills* [Finger Tapping Test – Right (FTT-R), Finger Tapping Test – Left (FTT-L)]; *verbal fluency* [Phonemic Fluency Task (PFT), Semantic Fluency Task (SFT)]. These cognitive domains have been consistently reported as vulnerable to HIV-related brain injury and are included in standard neurocognitive batteries for the diagnosis of HAND[1-5].

For each test, the primary outcome measure was selected according to established clinical practice and normative references. Specifically, total correct responses were used for DST-F/B, CBTT, SDMT, PFT, and SFT; completion time was used for TMT-A, TMT-B, and SCWT-T; error scores were used for SCWT-E; total number of taps was used for the FTT-R/L; and total recall scores were used for RAVLT-IR, RAVLT-DR, and ROCF-DR. Raw scores from each test were corrected for age, sex, and educational level using Italian normative data.

The autonomy in the activities of daily living was assessed by Instrumental Activities of Daily Living scale (IADL).

On the basis of the demographically-adjusted scores from neurocognitive tests and the IADL score, participants were diagnosed with HIV-associated neurocognitive disorder (HAND) as defined by the 2007 Frascati criteria[1].

Additionally, the raw scores from each test were converted into standardised *T*-scores based on the mean and the standard deviation (SD) in the entire study population [$T = 50 + 10\ (x - \text{mean/SD})$ or $T = 50 + 10\ (\text{mean/SD} - x)$ depending on whether a higher score corresponds to better or worse performance, respectively]. Domain-specific composite *T*-scores were calculated by averaging the *T*-scores of the individual tests defining the specific cognitive domain. The global cognitive composite

T-score was then obtained by averaging the domain-specific composite *T*-scores. Higher *T*-scores indicate better cognitive performance.

T-cell phenotypes

CD4 T-cells (CD4+), CD8 T-cells (CD8+), functionally-competent CD4 T-cells (CD4+CD127+), activated CD8 T-cells (CD8+CD38+), naïve CD4 T-cells (CD4+CD45RA+), and naïve CD8 T-cells (CD8+CD45RA+) were quantified in people with HIV on fresh whole blood by flow. Briefly, samples were prepared with the automated BD FACSDuet™ Sample Preparation System by mixing 50 µL of fresh whole blood and 40 µL of fluorescently-labelled antibodies cocktail (CD45–V500, CD4–APC-Cy7, CD8–PerCP-Cy5.5, CD127–PE, CD38–APC, CD45RA–PE-Cy7) in BD Trucount™ Tubes. After a 20-minute incubation at room temperature, red blood cells were lysed with BD FACS™ Lysing Solution. Samples were then acquired on a BD FACSLyric™ Flow Cytometry System, and fcs files analysed with BD FACSuite™ Clinical Application software. The gating strategy for a representative sample is shown in **Figure S1**.

Plasma biomarkers

Biomarkers of neuroaxonal damage [neurofilament light chain (NF-L)] and astrocyte activation [glial fibrillary acidic protein (GFAP)] were measured by Quanterix™ Simoa® Neurology 2-Plex B Kit on the Quanterix™ SR-X® Biomarker Detection System.

Biomarkers of inflammation [IFN-γ–induced protein 10 (IP-10), interleukin 6 (IL-6)], monocyte activation [soluble CD14 (sCD14)], gut barrier disruption [intestinal fatty acid binding protein (I-FABP), E-cadherin, zonulin] and microbial translocation [lipopolysaccharide-binding protein (LBP), endotoxin core antibodies IgG (EndoCAb IgG)] were measured by ELISA using commercially available kits (R&D Systems™ Quantikine® ELISA Kit Human CXCL10/IP-10, Human IL-6, Human FABP2/I-FABP; Hycult® Biotech ELISA Kit Human sCD14, Human LBP; Elabscience® ELISA Kit Human E-Cadherin, Human Zonulin; ELK Biotechnology ELISA Kit Human EndoCAb IgG). The optical density (OD) was measured by using PerkinElmer® EnSight™ Multimode Plate Reader.

Supplementary figures

Figure S1. Gating strategy for the identification of the T-cell phenotypes in a representative sample (generated with FlowJo v10).

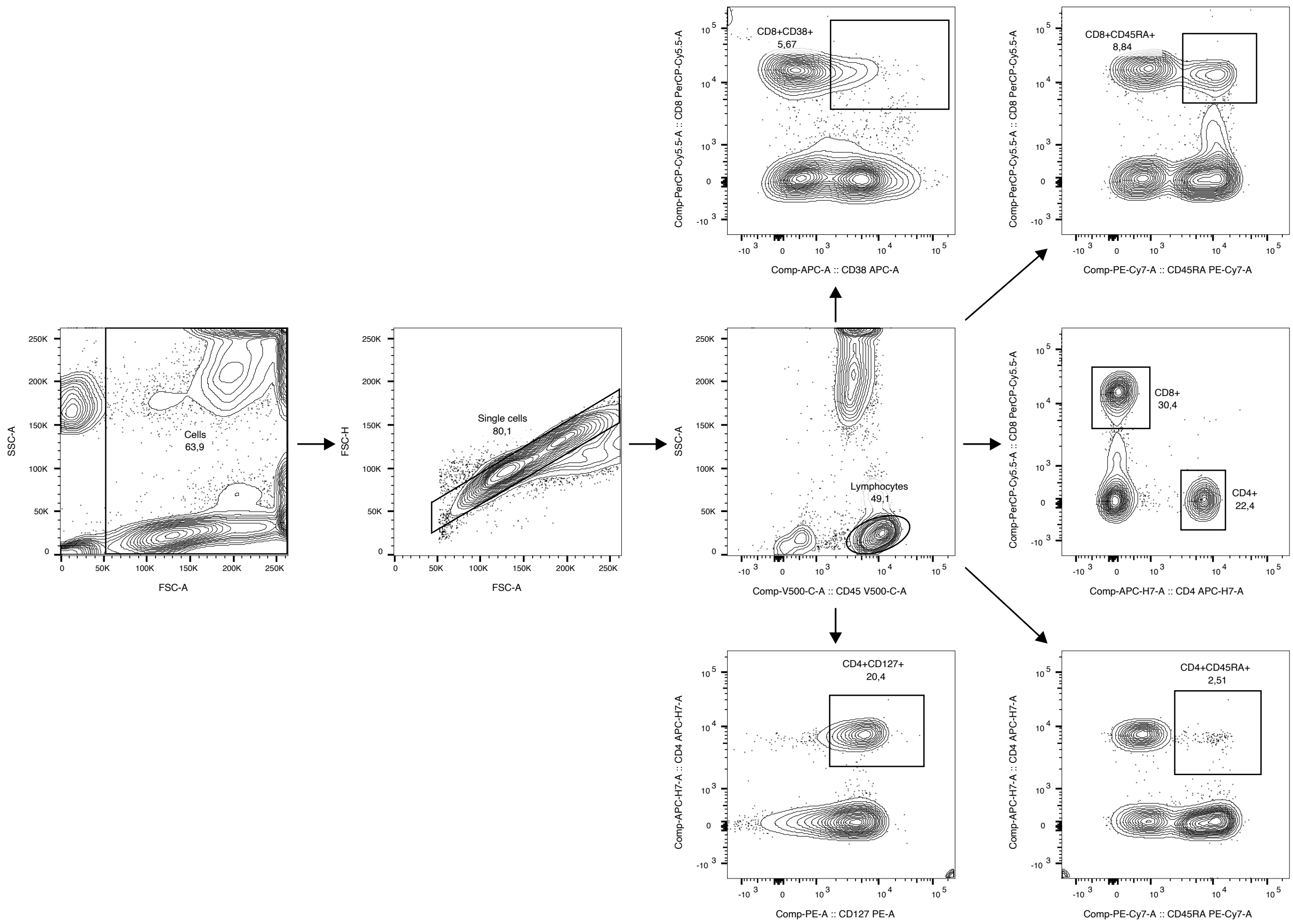

Figure S2. (**a–f**) Cognitive performances as measured by domain-specific composite *T*-scores in people with HIV (PWH) HAND+ (*n*=17) and PWH HAND– (*n*=23). *Circles*: individual values; *black bars*: median values; *boxes*: interquartile ranges; *statistical analysis*: Mann-Whitney test (P values <0.05 shown in red). (**g**) Correlations between domain-specific composite *T*-scores in PWH (*n*=40). *Red cells*: positive correlations (r>0); *statistical analysis*: Spearman's correlation test (* statistical significance at P<0.05).

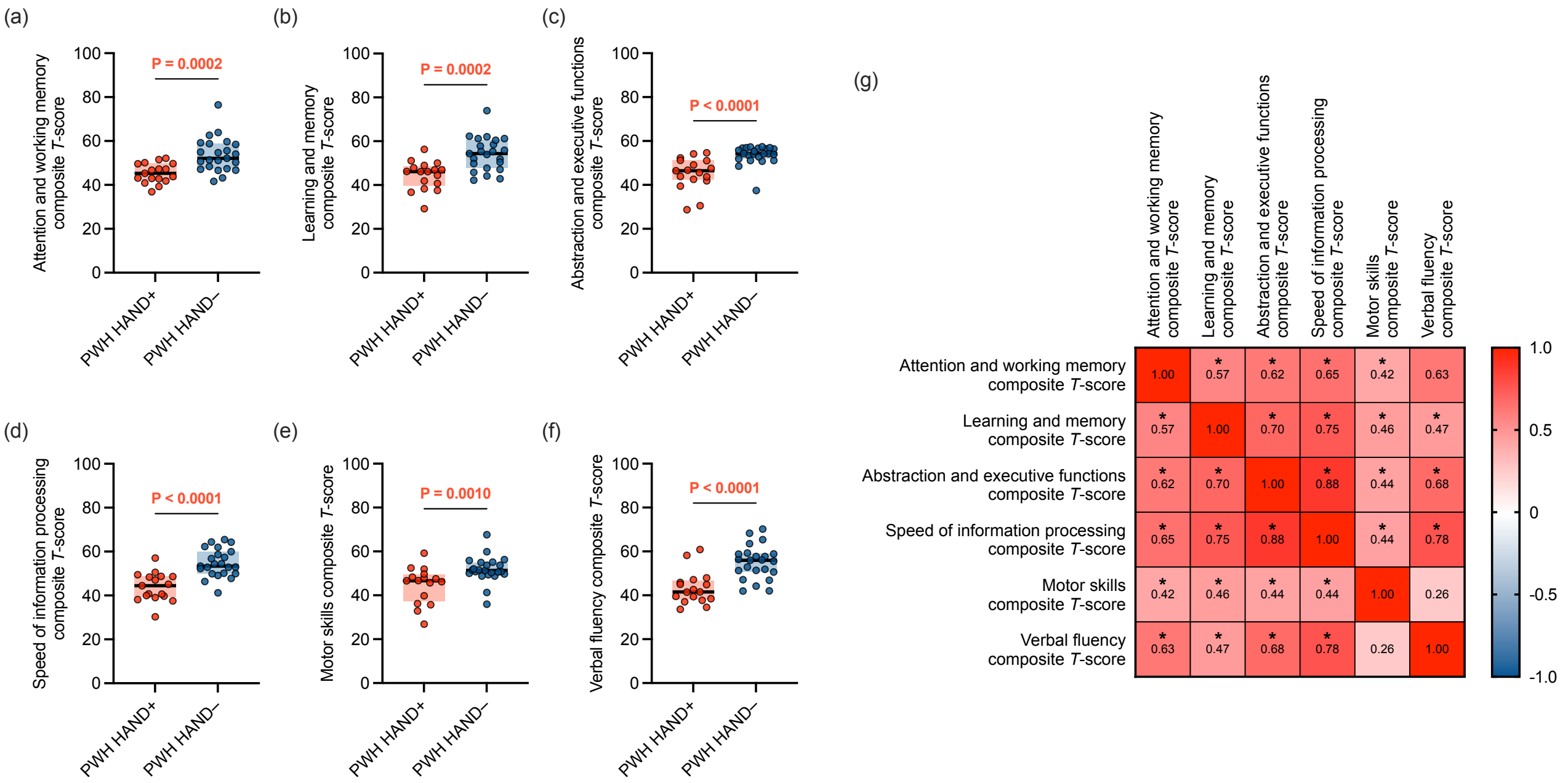

Figure S3. (**a–j**) Plasma biomarkers of neuroaxonal damage (NF-L), astrocyte activation (GFAP), inflammation (IP-10, IL-6), monocyte activation (sCD14), gut barrier disruption (I-FABP, E-cadherin, zonulin) and microbial translocation (LBP, EndoCAb IgG) in people with HIV (PWH) according to the ART regimen: NNRTI-based (*n*=19), PI-based (*n*=11), INSTI-based (*n*=8), and INSTI+PI-based (*n*=2). *Circles*: individual values; *black bars*: median values; *boxes*: interquartile ranges; *statistical analysis*: Kruskall-Wallis test with Dunn's multiple comparisons test.

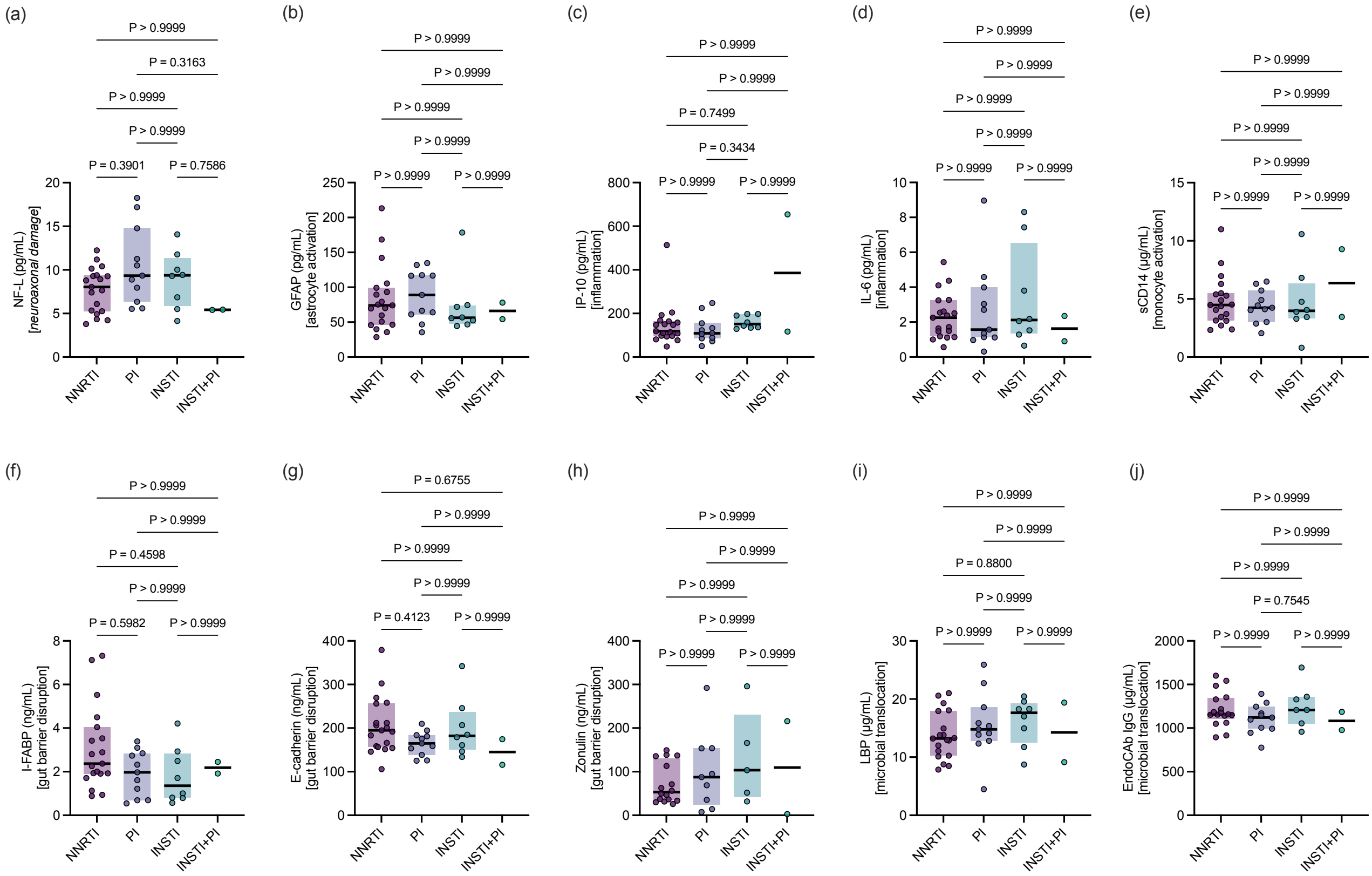

Figure S4. Correlations between plasma biomarkers of neuroaxonal damage (NF-L), astrocyte activation (GFAP), inflammation (IP-10, IL-6), monocyte activation (sCD14), gut barrier disruption (I-FABP, E-cadherin, zonulin) and microbial translocation (LBP, EndoCAb IgG) in the entire study population (*n*=50) including people with HIV (PWH) with HAND (*n*=17), PWH without HAND (*n*=23), and PWoH (*n*=10). *Blue cells*: negative correlations (ρ<0); *red cells*: positive correlations (ρ>0); *circles*: individual values; *black line*: fitted line*; statistical analysis*: Spearman's correlation test (* statistical significance at P<0.05).

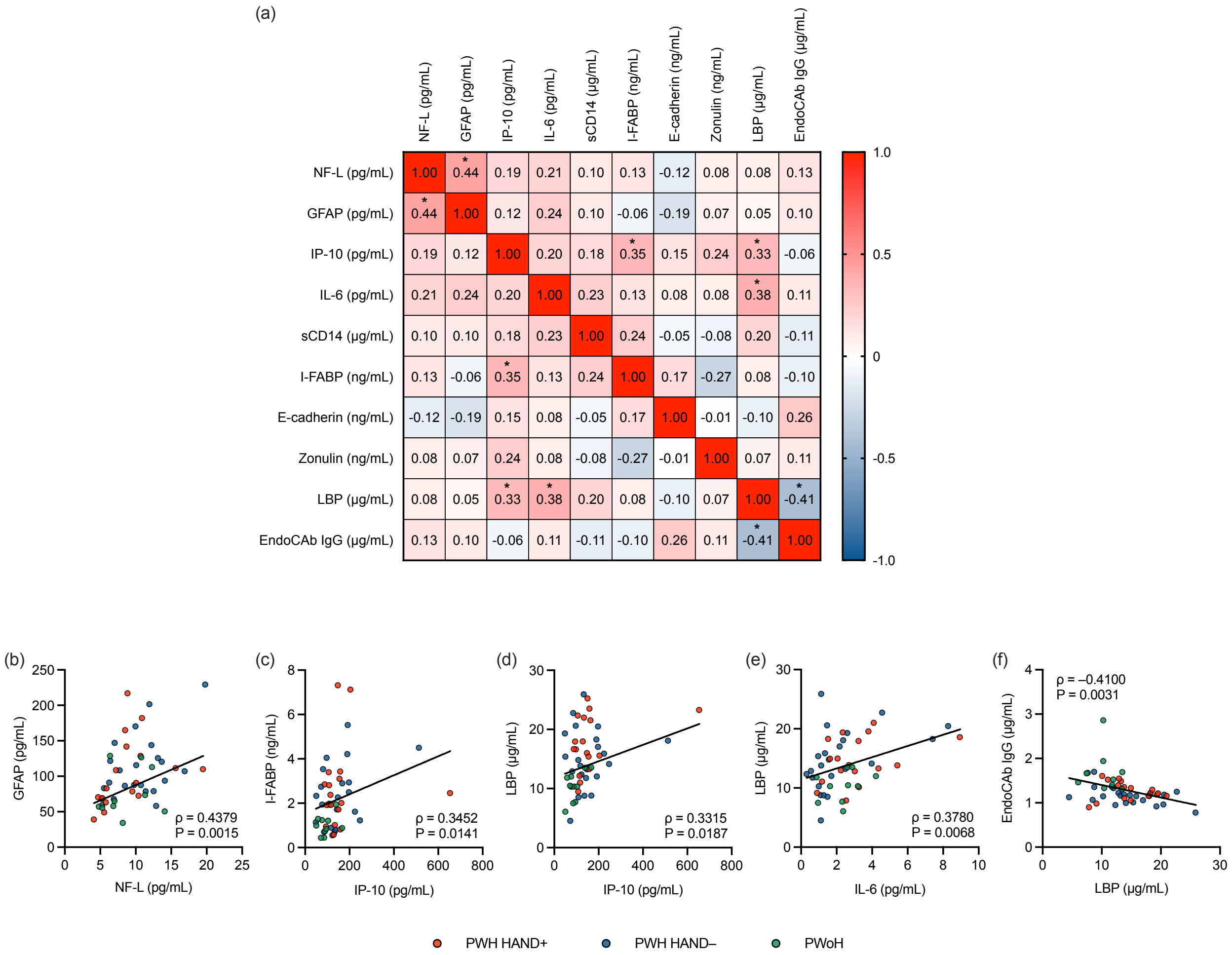

Supplementary tables

Table S1. Spearman's correlation analysis exploring the association between plasma biomarkers and the global/domain-specific composite *T*-scores (calculated by averaging the *T*-scores of the individual tests defining the specific cognitive domain) in people with HIV (*n*=40).

Biomarker	Spearman's correlation analysis		
	**ρ**	**95% CI**	**P value**
Global cognitive composite T-score			
NF-L (pg/mL)	-0.10	-0.40; 0.23	0.5531
GFAP (pg/mL)	-0.57	-0.75; -0.31	**0.0001**
IP-10 (pg/mL)	-0.05	-0.36; 0.28	0.7826
IL-6 (pg/mL)	-0.43	-0.66; -0.13	**0.0054**
sCD14 (µg/mL)	-0.40	-0.64; -0.09	**0.0100**
I-FABP (ng/mL)	0.19	-0.14; 0.48	0.2378
E-cadherin (ng/mL)	0.17	-0.16; 0.47	0.2900
Zonulin (ng/mL)	-0.06	-0.41; 0.30	0.7438
LBP (µg/mL)	-0.32	-0.58; -0.003	**0.0421**
EndoCAb IgG (ng/mL)	-0.44	-0.44; 0.22	0.4612
Attention and working memory composite T-score (DST-F, DST-B, CBTT, TMT-BA)			
NF-L (pg/mL)	0.05	-0.28; 0.36	0.7641
GFAP (pg/mL)	-0.42	-0.65; -0.11	**0.0077**
IP-10 (pg/mL)	0.04	-0.29; 0.35	0.8137
IL-6 (pg/mL)	-0.23	-0.61; 0.10	0.1547
sCD14 (µg/mL)	-0.38	-0.63; -0.07	**0.0147**
I-FABP (ng/mL)	0.38	0.07; 0.62	**0.0155**
E-cadherin (ng/mL)	0.19	-0.14; 0.48	0.2340
Zonulin (ng/mL)	-0.14	-0.47; 0.23	0.4531
LBP (µg/mL)	-0.09	-0.40; 0.24	0.5789
EndoCAb IgG (ng/mL)	-0.19	-0.50; 0.15	0.2483
Learning and memory composite T-score (RAVLT-IR, RAVLT-DR, ROCF-DR)			
NF-L (pg/mL)	-0.15	-0.45; 0.18	0.3577
GFAP (pg/mL)	-0.54	-0.74; -0.27	**0.0003**
IP-10 (pg/mL)	-0.03	-0.35; 0.29	0.8640
IL-6 (pg/mL)	-0.29	-0.56; 0.03	0.0668
sCD14 (µg/mL)	-0.21	-0.50; 0.11	0.1835
I-FABP (ng/mL)	0.27	-0.05; 0.55	0.0865
E-cadherin (ng/mL)	0.16	-0.17; 0.45	0.3355
Zonulin (ng/mL)	0.14	-0.23; 0.47	0.4568
LBP (µg/mL)	-0.31	-0.58; 0.01	**0.0494**
EndoCAb IgG (ng/mL)	-0.006	-0.34; 0.33	0.9733
Abstraction and executive functions composite T-score (ROCF-C, TMT-B, SCWT-E)			
NF-L (pg/mL)	-0.20	-0.49; 0.13	0.2226
GFAP (pg/mL)	-0.48	-0.70; -0.19	**0.0016**
IP-10 (pg/mL)	-0.13	-0.43; 0.20	0.4226
IL-6 (pg/mL)	-0.51	-0.71; -0.22	**0.0009**
sCD14 (µg/mL)	-0.31	-0.57; 0.01	0.0530
I-FABP (ng/mL)	-0.0004	-0.32; 0.32	0.9982
E-cadherin (ng/mL)	0.19	-0.13; 0.48	0.2313
Zonulin (ng/mL)	-0.06	-0.41; 0.30	0.7257
LBP (µg/mL)	-0.36	-0.61; -0.05	**0.0212**
EndoCAb IgG (ng/mL)	-0.22	-0.52; 0.12	0.1849
Speed of information processing composite T-score (TMT-A, SCWT-T, SDMT)			
NF-L (pg/mL)	-0.18	-0.47; 0.15	0.2758
GFAP (pg/mL)	-0.57	-0.75; -0.31	**0.0001**
IP-10 (pg/mL)	-0.04	-0.36; 0.28	0.7959
IL-6 (pg/mL)	-0.50	-0.71; -0.21	**0.0011**

sCD14 (µg/mL)	-0.37	-0.62; -0.06	**0.0176**
I-FABP (ng/mL)	0.11	-0.21; 0.42	0.4812
E-cadherin (ng/mL)	0.24	-0.08; 0.52	0.1321
Zonulin (ng/mL)	-0.05	-0.40; 0.31	0.7788
LBP (µg/mL)	-0.40	-0.64; -0.09	**0.0112**
EndoCAb IgG (ng/mL)	-0.04	-0.37; 0.29	0.7966
Motor skills composite T-score (FTT-R, FTT-L)			
NF-L (pg/mL)	0.13	-0.21; 0.43	0.4391
GFAP (pg/mL)	-0.43	-0.66; -0.13	**0.0057**
IP-10 (pg/mL)	0.16	-0.17; 0.46	0.3161
IL-6 (pg/mL)	0.06	-0.27; 0.38	0.7244
sCD14 (µg/mL)	-0.32	-0.58; 0.01	0.0502
I-FABP (ng/mL)	0.06	-0.27; 0.38	0.7226
E-cadherin (ng/mL)	0.19	-0.15; 0.48	0.2532
Zonulin (ng/mL)	0.14	-0.23; 0.48	0.4291
LBP (µg/mL)	-0.01	-0.34; 0.31	0.9337
EndoCAb IgG (ng/mL)	-0.21	-0.51; 0.13	0.2120
Verbal fluency composite T-score (PFT, SFT)			
NF-L (pg/mL)	0.004	-0.32; 0.32	0.9789
GFAP (pg/mL)	-0.30	-0.56; 0.02	0.0620
IP-10 (pg/mL)	-0.20	-0.49; 0.12	0.2081
IL-6 (pg/mL)	-0.43	-0.66; -0.13	**0.0053**
sCD14 (µg/mL)	-0.34	-0.59; -0.20	**0.0331**
I-FABP (ng/mL)	0.09	-0.24; 0.40	0.5869
E-cadherin (ng/mL)	0.10	-0.23; 0.41	0.5393
Zonulin (ng/mL)	-0.34	-0.62; 0.02	0.0562
LBP (µg/mL)	-0.28	-0.55; 0.04	0.0763
EndoCAb IgG (ng/mL)	-0.13	-0.45; 0.21	0.4260

Legend: *NF-L*: neurofilament light chain (neuroaxonal damage); *GFAP*: glial fibrillary acidic protein (astrocyte activation); *IP-10*: interferon-γ inducible protein 10 (inflammation); *IL-6*: interleukin 6 (inflammation); *sCD14*: soluble cluster of differentiation 14 (monocyte activation); *I-FABP*: intestinal fatty acid binding protein (gut barrier disruption); *E-cadherin* (gut barrier disruption); *zonulin* (gut barrier disruption); *LBP*: lipopolysaccharide binding protein (microbial translocation); *EndoCAb*: endogenous endotoxin-core antibody IgG; *DST-F*: Digit Span Test – Forward; *DST-B*: Digit Span Test – Backward; *CBTT*: Corsi Block Tapping Task; *TMT-BA*: Trail Making Test – part BA; *RAVLT-IR*: Rey Auditory Verbal Learning Test – Immediate Recall; *RAVLT-DR*: Rey Auditory Verbal Learning Test – Delayed Recall; *ROCF-DR*: Rey-Osterrieth Complex Figure test – Delayed Recall; *ROCF-C*: Rey-Osterrieth Complex Figure test – Copy; *TMT-B*: Trail Making Test – part B; *SCWT-E*: Stroop Colour and Word Test – Errors; *TMT-A*: Trail Making Test – part A; *SCWT-T*: Stroop Colour and Word Test – Time; *SDMT*: Symbol Digit Modalities Test; *FTT-R*: Finger Tapping Test – Right; *FTT-L*: Finger Tapping Test –Left; *PFT*: Phonemic Fluency Task; *SFT*: Semantic Fluency Task

Table S2. Univariable and multivariable linear regression analysis exploring the association between plasma biomarkers (independent variables) and the global/domain-specific composite *T*-scores (dependent variable, calculated by averaging the *T*-scores of the individual tests defining the specific cognitive domain) in people with HIV (*n*=40).

**Biomarker**	**Univariable analysis**			**Multivariable analysis***		
	**β coefficient**	**95% CI**	**P value**	**β coefficient**	**95% CI**	**P value**
Global cognitive composite T-score						
NF-L (pg/mL)	-0.40	-1.01; 0.22	0.2023	0.14	-0.60; 0.89	0.6994
GFAP (pg/mL)	-0.09	-0.13; -0.05	**<0.0001**	-0.07	-0.12; -0.02	**0.0103**
IP-10 (pg/mL)	0.005	-0.01; 0.02	0.6040	0.01	-0.002; 0.03	0.0861
IL-6 (pg/mL)	-1.31	-2.30; -0.32	**0.0107**	-0.80	-1.85; 0.26	0.1332

sCD14 (µg/mL)	-0.97	-1.92; -0.02	**0.0448**	-0.69	-1.56; 0.18	0.1166
I-FABP (ng/mL)	0.56	-0.78; 1.91	0.4005	0.60	-0.57; 1.78	0.3039
E-cadherin (ng/mL)	0.02	-0.02; 0.06	0.2450	0.01	-0.02; 0.05	0.3622
Zonulin (ng/mL)	-0.02	-0.05; 0.01	0.2075	-0.005	-0.03; 0.02	0.7165
LBP (µg/mL)	-0.42	-0.88; 0.03	0.0678	-0.09	-0.56; 0.38	0.6971
EndoCAb IgG (ng/mL)	-0.003	-0.01; 0.009	0.5910	-0.005	-0.01; 0.005	0.3243
Attention and working memory composite T-score (DST-F, DST-B, CBTT, TMT-BA)						
NF-L (pg/mL)	0.08	-0.65; 0.81	0.8234	0.52	-0.45; 1.49	0.2852
GFAP (pg/mL)	-0.08	-0.13; -0.03	**0.0044**	-0.09	-0.17; -0.03	**0.0085**
IP-10 (pg/mL)	0.023	0.002; 0.04	**0.0346**	0.03	0.01; 0.05	**0.0053**
IL-6 (pg/mL)	-0.85	-2.06; 0.37	0.1682	-0.68	-2.1; 0.74	0.3391
sCD14 (µg/mL)	-1.14	-2.24; -0.043	**0.0422**	-0.92	-2.07; 0.22	0.1110
I-FABP (ng/mL)	1.42	-0.08; 2.92	0.0625	1.45	-0.04; 2.95	0.0567
E-cadherin (ng/mL)	0.02	-0.02; 0.07	0.2487	0.02	-0.02; 0.06	0.3560
Zonulin (ng/mL)	-0.02	-0.053; 0.02	0.3284	-0.009	-0.05; 0.03	0.6354
LBP (µg/mL)	-0.04	-0.59; 0.51	0.8826	0.21	-0.41; 0.82	0.5008
EndoCAb IgG (ng/mL)	-0.01	-0.02; 0.006	0.2642	-0.009	-0.02; 0.005	0.1934
Learning and memory composite T-score (RAVLT-IR, RAVLT-DR, ROCF-DR)						
NF-L (pg/mL)	-0.76	-1.53; 0.013	0.0537	0.05	-0.96; 1.05	0.9273
GFAP (pg/mL)	-0.11	-0.17; -0.05	**0.0004**	-0.07	-0.15; -0.001	**0.0470**
IP-10 (pg/mL)	0.003	-0.02; 0.03	0.8257	0.01	-0.01; 0.03	0.3371
IL-6 (pg/mL)	-0.85	-2.21; 0.51	0.2118	0.30	-1.16; 1.77	0.6754
sCD14 (µg/mL)	-0.62	-1.90; 0.65	0.3282	-0.52	-1.72; 0.68	0.3854
I-FABP (ng/mL)	1.14	-0.57; 2.85	0.1848	1.09	-0.47; 2.66	0.1635
E-cadherin (ng/mL)	0.02	-0.03; 0.07	0.3730	0.01	-0.03; 0.06	0.5018
Zonulin (ng/mL)	-0.006	-0.05; 0.04	0.7995	0.02	-0.023; 0.053	0.4159
LBP (µg/mL)	-0.46	-1.05; 0.14	0.1303	-0.01	-0.64; 0.62	0.9752
EndoCAb IgG (ng/mL)	0.0004	-0.01; 0.02	0.9587	-0.001	-0.01; 0.01	0.8768
Abstraction and executive functions composite T-score (ROCF-C, TMT-B, SCWT-E)						
NF-L (pg/mL)	-0.65	-1.28; -0.01	0.0459	0.004	-0.78; 0.78	0.9912
GFAP (pg/mL)	-0.09	-0.1377; -0.05	**0.0003**	-0.05	-0.11; 0.003	0.0643
IP-10 (pg/mL)	-0.002	-0.022; 0.019	0.8826	0.008	-0.01; 0.03	0.4051
IL-6 (pg/mL)	-2.00	-2.94; -1.06	**0.0001**	-1.46	-2.48; -0.44	**0.0065**
sCD14 (µg/mL)	-0.57	-1.62; 0.48	0.2766	-0.33	-1.26; 0.60	0.4799
I-FABP (ng/mL)	0.11	-1.33; 1.55	0.8795	0.007	-1.17; 1.32	0.9042
E-cadherin (ng/mL)	0.02	-0.02; 0.06	0.3615	0.01	-0.02; 0.04	0.5283
Zonulin (ng/mL)	-0.02	-0.05; 0.008	0.1482	-0.006	-0.03; 0.02	0.5951
LBP (µg/mL)	-0.52	-0.99; -0.04	**0.0344**	-0.13	-0.62; 0.36	0.6044
EndoCAb IgG (ng/mL)	-0.006	-0.02; 0.006	0.3004	-0.008	-0.02; 0.002	0.0997
Speed of information processing composite T-score (TMT-A, SCWT-T, SDMT)						
NF-L (pg/mL)	-0.69	-1.44; 0.07	0.0738	-0.07	-0.99; 0.85	0.8774
GFAP (pg/mL)	-0.11	-0.17; -0.06	**0.0001**	-0.08	-0.14; -0.01	**0.0195**
IP-10 (pg/mL)	0.004	-0.02; 0.03	0.7523	0.02	-0.005; 0.04	0.1397
IL-6 (pg/mL)	-1.80	-3.01; -0.60	**0.0045**	-1.13	-2.42; 0.16	0.0847
sCD14 (µg/mL)	-1.13	-2.32; 0.07	0.0639	-0.78	-1.86; 0.30	0.1501
I-FABP (ng/mL)	0.30	-1.39; 1.99	0.7221	0.31	-1.16; 1.77	0.6744
E-cadherin (ng/mL)	0.027	-0.02; 0.07	0.2470	0.02	-0.02; 0.06	0.3681
Zonulin (ng/mL)	-0.015	-0.06; 0.03	0.4556	0.004	-0.03; 0.04	0.8045
LBP (µg/mL)	-0.66	-1.21; -0.10	**0.0222**	-0.24	-0.82; 0.33	0.3931
EndoCAb IgG (ng/mL)	0.001	-0.01; 0.02	0.8614	-0.001	-0.01; 0.01	0.8739
Motor skills composite T-score (FTT-R, FTT-L)						
NF-L (pg/mL)	0.15	-0.62; 0.92	0.6915	0.65	-0.23; 1.53	0.1419
GFAP (pg/mL)	-0.09	-0.14; -0.04	**0.0017**	-0.07	-0.13; -0.01	**0.0251**
IP-10 (pg/mL)	0.01	-0.02; 0.03	0.5374	0.02	0.0002; 0.04	**0.0478**
IL-6 (pg/mL)	0.16	-1.30; 1.62	0.8246	0.76	-0.70; 2.23	0.2956
sCD14 (µg/mL)	-0.68	-1.82; 0.47	0.2381	-0.22	-1.28; 0.85	0.6827
I-FABP (ng/mL)	0.56	-1.02; 2.14	0.4758	0.75	-0.65; 2.16	0.2845
E-cadherin (ng/mL)	0.01	-0.03; 0.06	0.5023	0.01	-0.03; 0.05	0.6234
Zonulin (ng/mL)	-0.01	-0.04; 0.03	0.6371	0.005	-0.03; 0.04	0.7737
LBP (µg/mL)	-0.32	-0.87; 0.23	0.2464	-0.06	-0.62; 0.49	0.8239
EndoCAb IgG (ng/mL)	-0.0001	-0.01; 0.01	0.9824	-0.003	-0.01; 0.01	0.6447
Verbal fluency composite T-score (PFT, SFT)						
NF-L (pg/mL)	-0.31	-1.20; 0.58	0.4860	-0.13	-1.33; 1.07	0.8247
GFAP (pg/mL)	-0.063	-0.13; 0.01	0.0795	-0.04	-0.13; 0.05	0.3527
IP-10 (pg/mL)	-0.006	-0.03; 0.02	0.6428	-0.002	-0.03; 0.03	0.8829

IL-6 (pg/mL)	-1.91	-3.31; -0.51	**0.0088**	-2.02	-3.63; -0.41	**0.0154**
sCD14 (μg/mL)	-1.69	-3.00; -0.37	**0.0134**	-1.40	-2.76; -0.04	**0.0447**
I-FABP (ng/mL)	-0.007	-1.94; 1.93	0.9943	0.11	-1.81; 2.02	0.9117
E-cadherin (ng/mL)	0.02	-0.03; 0.07	0.4282	0.02	-0.04; 0.07	0.5515
Zonulin (ng/mL)	-0.05	-0.09; -0.005	**0.0296**	-0.04	-0.07; 0.006	0.0881
LBP (μg/mL)	-0.49	-1.15; 0.17	0.1397	-0.31	-1.06; 0.44	0.4080
EndoCAb IgG (ng/mL)	-0.005	-0.02; 0.01	0.5149	-0.007	-0.02; 0.01	0.3748

Legend: *NF-L*: neurofilament light chain (neuroaxonal damage); *GFAP*: glial fibrillary acidic protein (astrocyte activation); *IP-10*: interferon-γ inducible protein 10 (inflammation); *IL-6*: interleukin 6 (inflammation); *sCD14*: soluble cluster of differentiation 14 (monocyte activation); *I-FABP*: intestinal fatty acid binding protein (gut barrier disruption); *E-cadherin* (gut barrier disruption); *zonulin* (gut barrier disruption); *LBP*: lipopolysaccharide binding protein (microbial translocation); *EndoCAb*: endogenous endotoxin-core antibody IgG; *DST-F*: Digit Span Test – Forward; *DST-B*: Digit Span Test – Backward; *CBTT*: Corsi Block Tapping Task; *TMT-BA*: Trail Making Test – part BA; *RAVLT-IR*: Rey Auditory Verbal Learning Test – Immediate Recall; *RAVLT-DR*: Rey Auditory Verbal Learning Test – Delayed Recall; *ROCF-DR*: Rey-Osterrieth Complex Figure test – Delayed Recall; *ROCF-C*: Rey-Osterrieth Complex Figure test – Copy; *TMT-B*: Trail Making Test – part B; *SCWT-E*: Stroop Colour and Word Test – Errors; *TMT-A*: Trail Making Test – part A; *SCWT-T*: Stroop Colour and Word Test – Time; *SDMT*: Symbol Digit Modalities Test; *FTT-R*: Finger Tapping Test – Right; *FTT-L*: Finger Tapping Test –Left; *PFT*: Phonemic Fluency Task; *SFT*: Semantic Fluency Task.
* Adjusted for age, sex, and CD4 T-cell *nadir*

References

1. Antinori A, Arendt G, Becker JT, Brew BJ, Byrd DA, Cherner M, et al. **Updated research nosology for HIV-associated neurocognitive disorders**. *Neurology* 2007; 69(18):1789-1799.
2. Bai F, Iannuzzi F, Merlini E, Borghi L, Tincati C, Trunfio M, et al. **Clinical and viro-immunological correlates of HIV associated neurocognitive disorders (HAND) in a cohort of antiretroviral-naïve HIV-infected patients**. *AIDS* 2017; 31(2):311-314.
3. Bai F, Bono V, Borghi L, Bonazza F, Falcinella C, Vitaletti V, et al. **Association between tight junction proteins and cognitive performance in untreated persons with HIV**. *AIDS* 2024; 38(9):1292-1303.
4. Trunfio M, De Francesco D, Vai D, Medina C, Milesi M, Domini S, et al. **Screening Accuracy of Mini Addenbrooke's Cognitive Examination Test for HIV-Associated Neurocognitive Disorders in People Ageing with HIV**. *AIDS Behav* 2022; 26(7):2203-2211.
5. Trunfio M, Mighetto L, Napoli L, Atzori C, Nigra M, Guastamacchia G, et al. **Cerebrospinal Fluid CXCL13 as Candidate Biomarker of Intrathecal Immune Activation, IgG Synthesis and Neurocognitive Impairment in People with HIV**. *J Neuroimmune Pharmacol* 2023; 18(1-2):169-182.
